# Honey-mediated aggregation of soymilk proteins

**DOI:** 10.1016/j.heliyon.2020.e03673

**Published:** 2020-04-12

**Authors:** Yasuhiro Arii, Kaho Nishizawa

**Affiliations:** Department of Food Science and Nutrition, School of Human Environmental Sciences, Mukogawa Women's University, Nishinomiya, Hyogo 663-8558, Japan

**Keywords:** Food science, Food technology, Aggregation, Gluconic acid, Honey, Soymilk

## Abstract

Gluconic acid, the major organic acid in honey, is a partial hydrolysate of glucono-δ-lactone, typically used as a coagulant in preparing tofu. The present study aimed to examine the coagulation potential of five different types of honey at different concentrations, upon addition to soymilk. In some samples, aggregates formed in the upper layer at a higher honey concentration, while in others, aggregates precipitated at an intermediate honey concentration. Both phenomena were reproduced by adding different mixtures of glucono-δ-lactone and glucose, indicating that gluconic acid concentration and total sugar content of honey can trigger soymilk coagulation. Interestingly, honeys with a high concentration of gluconic acid showed a low total sugar content. Furthermore, in a trial product, the mixture of blended honey with soymilk was determined to be pasty. Our results indicate that honey can coagulate soymilk, which may provide a new and convenient method to prepare soymilk-based industrial products.

## 1 Introduction

Honey has been used as a food and medicinal item since ancient times. To data, honey is still used in various food products because of its characteristic flavour, taste, and texture. Honey has recently attracted attention as a functional food, in particular, due to the health benefits associated with natural and/or organic-based honey production. Honey contains numerous carbohydrates, primarily comprising fructose and glucose and respective secondary oligosaccharides (Tewari and Irudayaraj, 2004), and small quantities of organic acids, such as flavonoids, polyphenols, carotenoid-like substrates, Maillard reaction products, vitamins, minerals, and water (Mato et al., 2006; Machado De-Melo et al., 2018). The exact composition of honey dependents on the source, such as nectar and pollen, the collecting time across the year, as well as honeybee phenotypes along with other environmental factors (Alvarez-Suartez et al., 2014). The organic acids greatly contribute to the properties and functions of honey despite being present in small quantities (<0.5%). Gluconic acid (GA) is the major organic acid (>79%) in honey (Mato et al., 2006) and is mainly produced by the enzymatic oxidation of glucose in honeybee (Carina et al., 2014). Although the enzymatic oxidation is inhibited by a low quantity of available water and the acidic pH in honey, the activity is resumed when the honey is diluted in water (Carina et al., 2014).

In the industrial preparation of tofu, glucono-δ-lactone (GDL) is added as a coagulant in soymilk (Nakayama et al., 1965). GDL is a lactone of GA that is spontaneously hydrolysed in water to form GA (Sawyer and Bagger, 1959), thereby reducing the pH (Chen et al., 2016). This pH reduction leads to the formation of a protein gel by decreasing electrostatic repulsion between proteins in soymilk (Cavallieri and da Cunha, 2008). The hydrolysis is enhanced by heating (Pocker and Green, 1972). Besides, GDL-coagulated tofu has higher breaking stress than that of CaSO_4_-coagulated tofu (Chen et al., 2002). These findings indicate the possibility that honey with suitable GA content can be used as an alternative to GDL addition to inducing protein aggregation in soymilk, thereby facilitating the preparation of sweets with the functional properties of both soybean and honey.

In the present study, we tested this hypothesis by assessing the effects of different kinds of honey (acacia, bindweed, buckwheat, coffee, and blended) on soymilk proteins. We also determined the specific total sugar content and GA concentration of the different kinds of honey. Furthermore, a trial product was made by mixing soymilk with the blended honey, and its texture was evaluated.

## 2 Materials and methods

### 2.1 Materials

Acacia (Lot No. 5K23), bindweed (Lot No. 4J02), buckwheat (Lot No. 6K02), and coffee (Lot No. 6J03) jars of honey were generously provided by Yamada Bee Farm (Okayama, Japan). Blended honey was purchased from Kato Brothers Honey Co., Ltd (Tokyo, Japan). The colour of the honey was classified into two groups, namely sunny-yellow for acacia and blended honeys, and blackish-brown for buckwheat, bindweed, and coffee honeys. Soymilk was purchased from Sujahta Meiraku (Aichi, Japan). GDL was generously provided by Ako Kasei Co., Ltd (Hyogo, Japan). GA assay kit was purchased from Roche Diagnostics GmbH (Mannheim, Germany). Other chemicals were purchased from Wako Pure Chemical Industries, Ltd. (Osaka, Japan).

### 2.2 Preparation of diluted honey solutions and GDL/glucose mixtures

Since honey has a high viscosity, it is challenging to measure an exact amount of honey to be added to soymilk. Therefore, to maintain experimental reproducibility, diluted honey solutions were prepared by adding honey to distilled water at various concentrations (w/w). To analyse the relationship between sugar content and upper layer aggregation, in the reproductive experiments of aggregation behaviours, GDL/glucose mixtures were freshly prepared at a concentration of 0.5% (w/w) GDL and 65% (w/w) glucose or 0.3% (w/w) GDL and 71% (w/w) glucose, and then incubated at 60 °C for complete dissolution. To analyse the effect of the sugar concentration on aggregation in the upper layer, diluted GDL/glucose solutions were also prepared at various concentrations of GDL and glucose.

### 2.3 Detection of coagulation

In the experiment of tofu formation, the coagulation is conventionally assessed by adding a coagulant to soymilk (Guo and Ono, 2005; Arii and Takenaka, 2013, 2014; Chen et al., 2016; Hsia et al., 2016; Arii and Nishizawa, 2018). We assessed soymilk coagulation according to the methods previously described by Arii and Takenaka (2013), with minor modifications. Soymilk was incubated at 85 °C for 5 min. The temperature was selected according to published data (Yang and James, 2012). Diluted honey solution or diluted GDL/glucose solution was added to an equal weight of the incubated soymilk with thorough mixing, after which it was incubated at 85 °C for 60 min. Soymilk was added to an equal volume of solutions containing different honey concentrations. The mixture was incubated on ice for 60 min, and separated into liquid and solid phases via centrifugation at 8,000 × *g* for 10 min at 4 °C. After that, the mixture was visually observed. A part of the liquid phase was used to determine the pH and subjected to SDS-polyacrylamide gel electrophoresis (SDS-PAGE) analysis.

### 2.4 pH measurement

The pH of the liquid phase and diluted honey solution was measured using a compact pH meter (LAQUA twin, HORIBA, Ltd, Kyoto, Japan). Data are presented as the mean ± standard deviation values from three independent experiments.

### 2.5 SDS-PAGE

To confirm whether soy proteins aggregated upon the addition of honey in soymilk, proteins in the mixture were analysed by SDS-PAGE. The mixtures were treated as indicated above. To analyse soymilk proteins, distilled water was first added to soymilk instead of honey. In contrast, to analyse honey proteins, honey was added to distilled water as a substitute for soymilk. One volume of the liquid phase was diluted in 39 volumes of distilled water. SDS-PAGE was carried out by the methods of Nishizawa and Arii (2016). The molecular weight standard was purchased from Life Technologies (Tokyo, Japan).

### 2.6 Determination of total sugar content

The total sugar content of honey was determined according to the method of DuBois et al. (1956) with certain modifications. Glucose was used as the standard solution to determine relative sugar concentrations. Honey was diluted 2 × 10^4^-fold with distilled water. The diluted honey was mixed thoroughly with an equal volume of 5% phenol and with 5 volumes of concentrated sulphuric acid. The mixture was left for 10 min and then cooled at 25 °C for 10 min. The mixture's absorbance was measured at 490 nm.

### 2.7 Determination of GA concentration

GA concentration was determined using the GA assay kit according to the manufacturer's instructions, with certain modifications. For the standard solution, 0.67 mg/mL sodium gluconate was prepared. Honeys were diluted in distilled water to prepare five samples containing 10% coffee honey, 10% buckwheat honey, 20% acacia honey, 20% bindweed honey, and 20% blended honey, respectively. The samples were mixed with 0.5 volumes of a solution containing triethanolamine buffer (pH 7.6), nicotinamide adenine triphosphate (NADP), and adenosine triphosphate, and 0.01 volume of a solution containing 6-phosphogluconate dehydrogenase. Each mixture was incubated at 20 °C for 5 min, and then its absorbance (*A*_1_) was measured at 365 nm. The mixture was further incubated for 20 min after adding 0.01 volume of gluconate kinase suspension, and then its absorbance (*A*_2_) was measured at 365 nm. For the blank sample, only distilled water was used. The difference in absorbance (Δ*A*) was calculated as follows:

(1) Δ*A* = (*A*_2_ – *A*_1_) – (*A*_2b_ – *A*_1b_)

where *A*_2b_ and *A*_1b_ indicate the absorbance of the mixtures for the blank sample. GA concentration (*c*) was calculated as follows:

(2) *c* = (*V* × *M* × Δ*A*) / (*ε* × *d* × *v* × 1000)

where *V*, *M*, *ε*, *d*, and *v* represent the volume of the reaction mixture, the molecular mass of GA, the molecular extinction coefficient of NADP, light path length, and volume of the sample, respectively. Data represent the mean ± standard deviation values from three independent experiments.

### 2.8 Preparation of a trial product

Blended honey was mixed well with soymilk (honey concentration, (w/w) 43%) at 25 °C. The mixture was poured into a jam bottle (Type 1, AS ONE, Osaka, Japan) with an inside diameter of 40 mm. The weight of the poured mixture was 25 g. The bottled mixture was steamed for 60 min at low heat, followed by incubation on ice for 60 min. The product was stored at 4 °C overnight.

### 2.9 Measurement of the product texture

Hardness, cohesiveness, adhesiveness, and thickness of the trial product were measured according to the method of texture profile analysis test (Pons and Fiszman, 1996; Rosenthal, 2010) with some modifications in the texture mode, using a rheometer (RE2-33005S, Yamaden Co. Ltd., Tokyo, Japan). The jam bottle used, had the following measurements and conditions: plunger diameter, 20 mm; clearance, 5 mm; test speed, 1 mm/s; temperature, 20 °C. Data represent the mean ± standard deviation values from three independent experiments.

### 2.10 Statistical analyses

To compare GA concentration and total sugar content, a one-way analysis of variance was performed for multiple-group comparisons, followed by the Tukey-Kramer test for *posthoc* analysis with Aabel 3 (Hulinks, Tokyo, Japan). For the pH comparison, Student's *t*-test was performed. Differences with *p* values <0.05 were considered statistically significant.

## 3 Results and discussion

### 3.1 Precipitate formation upon addition of honey

**Figure 1:**
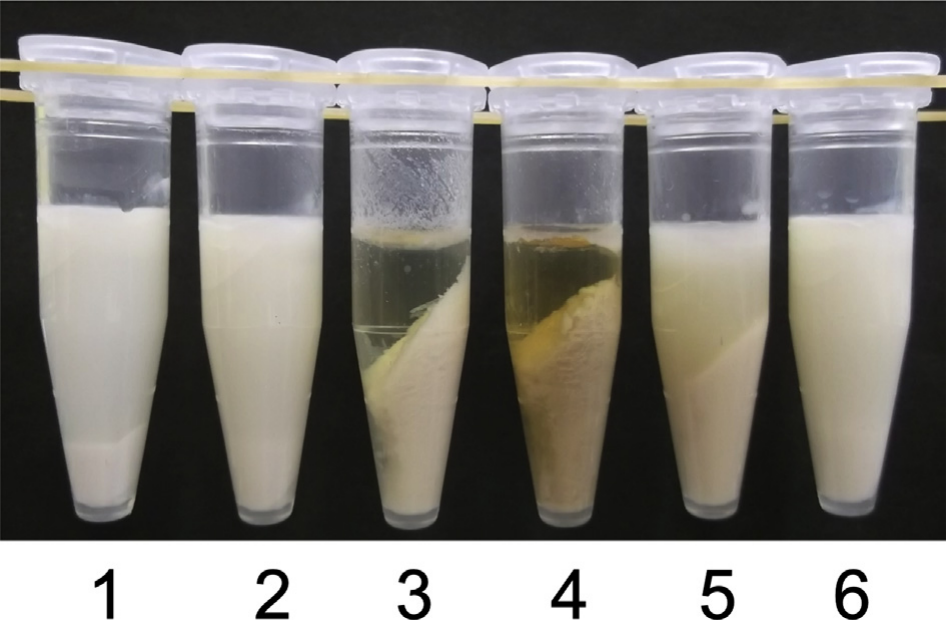
Protein precipitation upon the addition of honey. As a control (1), distilled water was added to soymilk. Acacia (2), coffee (3), buckwheat (4), bindweed (5), and blended (6) kinds of honey at different concentrations were added to soymilk at a final concentration of 25%.

Since the actual composition of honey differs between honey samples from different sources (Alvarez-Suartez et al., 2014), five different honey samples were selected to analyse the respective abilities further to induce soymilk proteins aggregation. To investigate the ability of the honey to trigger soymilk precipitate, diluted honey (50%) was added to an equal volume of soymilk at a final concentration of 25% (Figure 1). A small amount of precipitate was observed in the mixtures containing acacia and blended kinds of honey, whereas coffee and buckwheat honeys produced a large amount of precipitate (Figure 1). Besides, a large amount of precipitate was observed in the bindweed honey mixture; however, the supernatant remained turbid (Figure 1). Differences in precipitation indicate that the type of honey influenced the tendency for precipitation.

### 3.2 Concentration-dependent aggregation by honey

**Figure 2:**
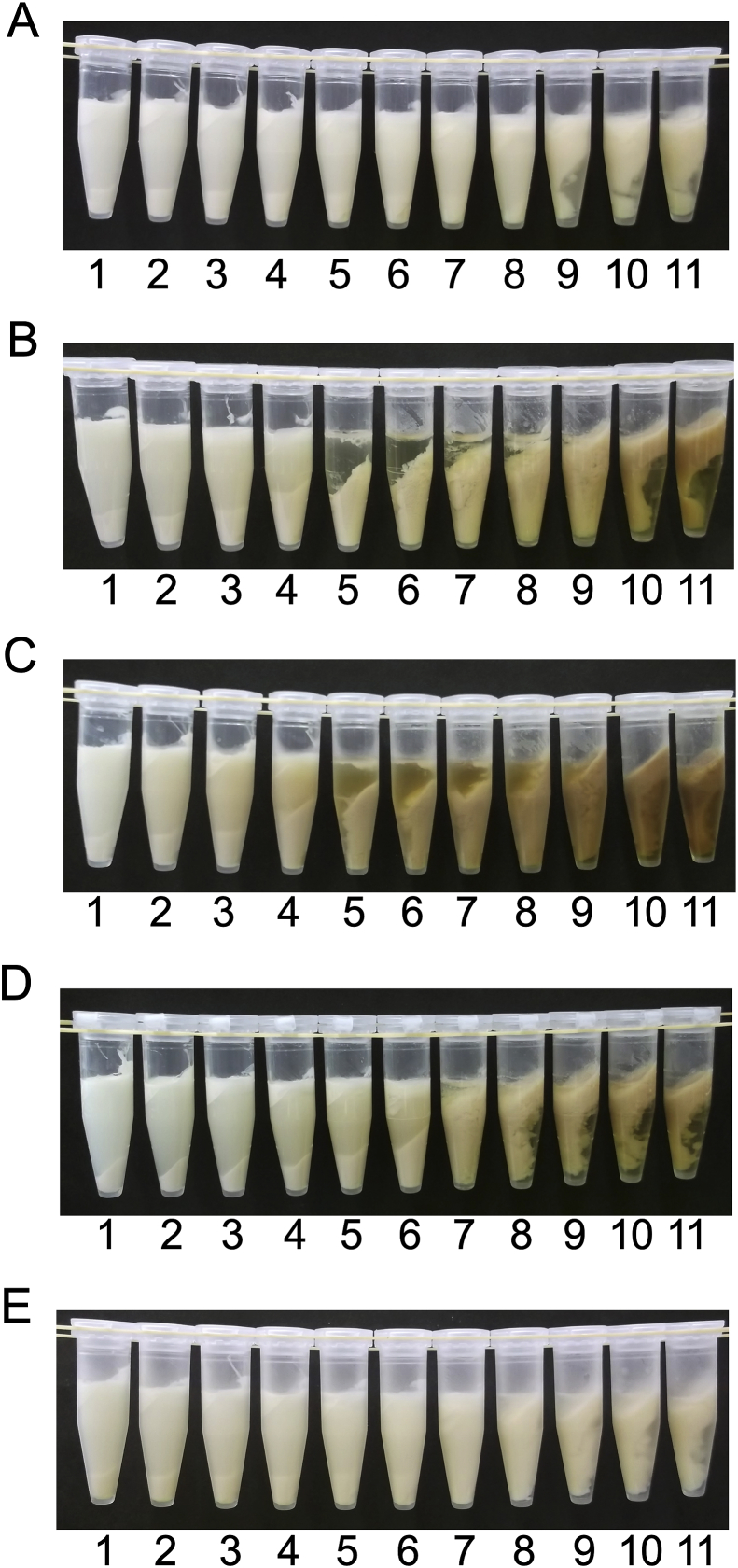
Concentration-dependent protein aggregation by honey. Acacia (A), coffee (B), buckwheat (C), bindweed (D), and blended (E) honeys at different concentrations were added to soymilk at a final concentration of 0% (1), 5% (2), 10% (3), 15% (4), 20% (5), 25% (6), 30% (7), 35% (8), 40% (9), 45% (10), and 50% (11).

Adding honey at various concentrations would also induce precipitation to a different extent. To investigate the effect of concentration of honey on precipitation of soymilk, acacia, coffee, buckwheat, bindweed, and blended honeys ranging from 0-50% (w/w) in concentration were mixed with soymilk and separated via centrifugation (Figure 2). The precipitation did not increase markedly in the acacia honey mixture; however, aggregation was observed in the upper layer at a concentration of 40% and above (Figure 2A). In coffee (Figure 2B) and buckwheat (Figure 2C) honey mixtures, precipitation increased when honey was added at the concentration of 15–35%; precipitates did not clear, and aggregates were observed in the upper layer with above 40% honey concentration. In the bindweed honey mixture (Figure 2D), precipitation increased markedly with 20–30% honey concentration, and upon increasing the honey concentration to more than 35%, aggregates were observed in the upper layer instead of as precipitates. In the blended honey mixture (Figure 2E), precipitation was indistinctly observed when honey was added at the concentration of 35–40%; aggregates were observed in the upper layer with more than 45% honey. From the results of Figure 2, the honey concentration ranges at which aggregation was observed are summarized in Table 1. Together, these results indicate that the aggregation depends on honey concentration and type.

**Table 1:** Honey concentration range resulting in aggregation.

	Honey concentration range^1^ (%)	
	Lower layer	Upper layer
Acacia	ND	40–50
Coffee	15–35	40–50
Buckwheat	15–35	40–50
Bindweed	20–30	35–50
Blended	35–40	45–50

1 Honey concentration ranges are summarized from the results of Figure 2.

### 3.3 Aggregation of proteins in soymilk

**Figure 3:**
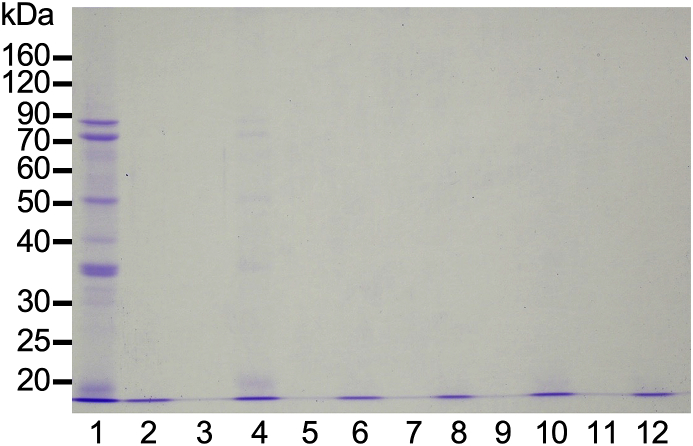
Protein aggregation analysis by SDS-PAGE. Acacia (lanes 3 and 4), coffee (lanes 5 and 6), buckwheat (lanes 7 and 8), bindweed (lanes 9 and 10), and blended (lanes 11 and 12) honeys at different concentrations were added to an equal volume of distilled water (lanes 3, 5, 7, 9, and 11) or soymilk (lanes 4, 6, 8, 10, and 12) at a final honey concentration of 50%. Distilled water (lane 1) or 0.5% GDL (lane 2) was also added to an equal volume of soymilk. The final GDL concentration was 0.25%. Mixtures were incubated and centrifuged. A part of the liquid phase was used for SDS-PAGE analysis.

In tofu formation, soy protein aggregation is commonly triggered by adding a coagulant (Arii and Takenaka, 2013). To determine whether soy proteins aggregated upon the addition of honey in soymilk, the mixture with a honey concentration of 50% was separated into a liquid and solid phase via centrifugation. As shown in Figure 2, since the separation of these phases, was incomplete, it was difficult to analyse only the precipitate. Thus, the liquid phase was analysed via SDS-PAGE (Figure 3). Most soymilk proteins were diminished in the liquid phase by adding GDL. The liquid phase contained small amounts of proteins in each honey mixture, and no proteins were detected in any of the mixtures containing diluted honey. These results indicate that proteins in soymilk aggregated upon the addition of honey.

### 3.4 Change in soymilk pH upon addition of honey

**Figure 4:**
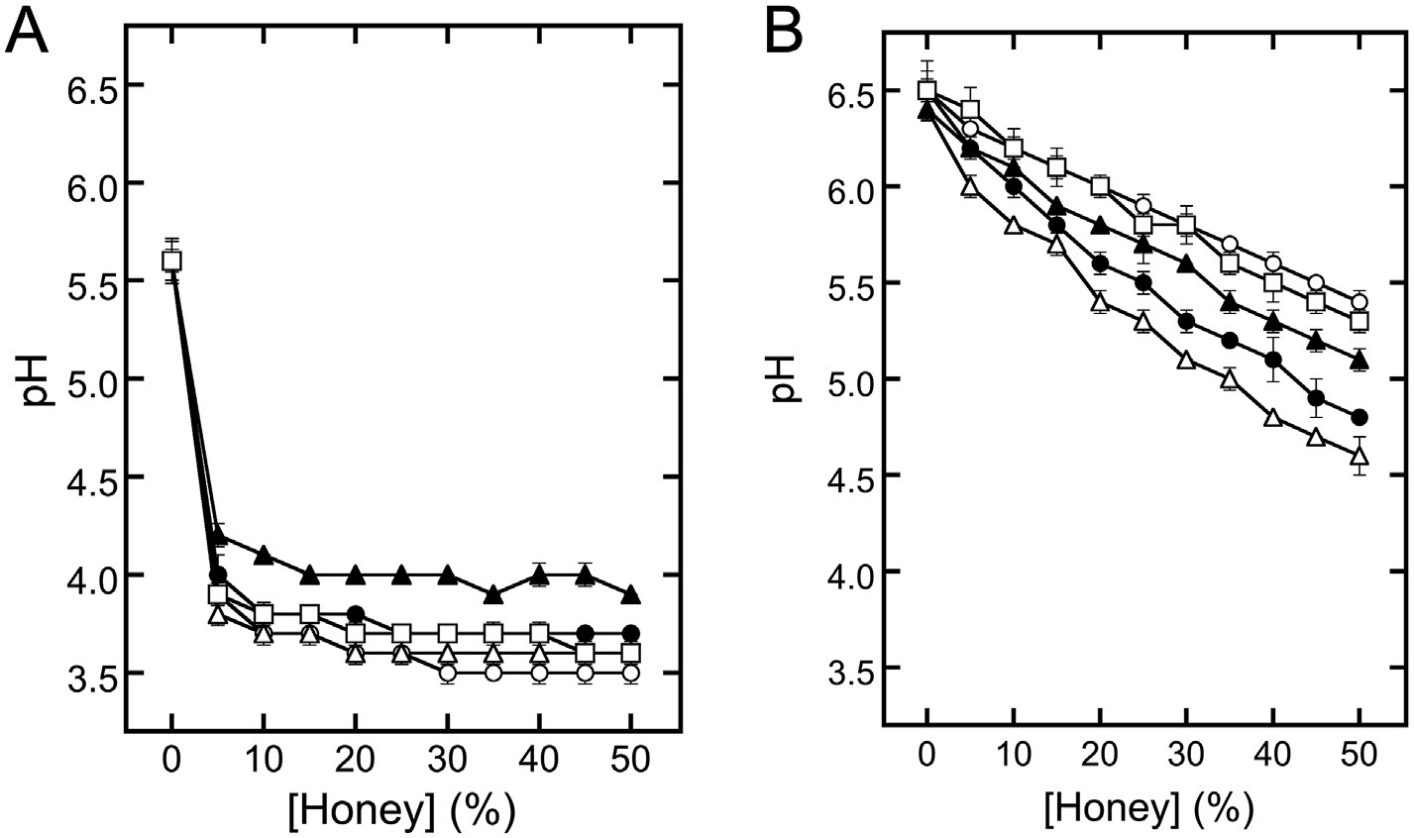
Changes in soymilk pH upon the addition of honey. Honey was mixed with distilled water (A) or soymilk (B) at various concentrations. The mixture was incubated and centrifuged. The pH of the supernatant was measured. Open circles, closed circles, open triangles, closed triangles, and open squares indicate acacia, coffee, buckwheat, bindweed, and blended kinds of honey, respectively.

In GDL-coagulated tofu formation, pH reduction decreases electrostatic repulsion between soymilk proteins, ultimately leading to the formation of a protein gel (Cavallieri and da Cunha, 2008). To investigate the effect of honey on soymilk pH, each type of honey was separately mixed with distilled water (Figure 4A) or soymilk (Figure 4B) at different concentrations. The pH values of the mixtures with distilled water drastically decreased even at a honey concentration of 5% (*p* < 0.01). At honey concentration of 50%, the pH values were low and in the following order: acacia honey (pH 3.5) < buckwheat honey (pH 3.6) ≈ blended honey (pH 3.6) < coffee honey (pH 3.7) < bindweed honey (pH 3.9) (Figure 4A). This order could be due to the concentration of organic acids in honey. The primary organic acid in honey is GA, which accounts for more than 79% of organic acid content (Mato et al., 2006). The pH values of the mixtures with soymilk gently decreased with an increase in honey concentration. At a honey concentration of 50%, the values were low and in the following order: buckwheat honey (pH 4.6) < coffee honey (pH 4.8) < bindweed honey (pH 5.1) < blended honey (pH 5.3) < acacia honey (pH 5.4) (Figure 4B). Different honeys have different organic acids in small amounts (Mato et al., 2006), which have different, but specific acid strengths; this may have influenced the pH of the mixture of honey with soymilk. The change in the order of pH may have resulted from differences in the composition of minor organic acids in each honey.

### 3.5 Association between pH changes and protein aggregation

We observed that different concentrations of each honey induced protein aggregation (Figure 2). The concentrations were approximately 40%, 30%, 20%, 20%, and 35% for acacia, bindweed, buckwheat, coffee, and blended honey mixtures, respectively (Figure 2). The pH values are summarized in Table 2 from the results of Figures 2 and 4B. Notably, protein aggregation was induced at a similar pH of 5.4–5.6 of the different types of honey (Table 2). The isoelectronic point of soy protein isolates is pH 5.53 ± 0.07 (Tsukada et al., 2006), and gelation of soymilk is induced at approximately pH 5.8 during acid-induced gelation, using GDL (Ringgenberg et al., 2013). These findings support that honey might trigger protein aggregation upon attainment of the isoelectronic point. The pH values (5.4–5.6) exhibited herein are slightly lower than those reported (pH 5.8) by Ringgenberg et al. (2013). This minor pH difference may have resulted from the high viscosity of honey and the visual assessment of aggregation. Protein aggregation in the upper layer was evaluated for the association between honey concentrations and pH values (Table 2). Honey concentrations and pH values were 40% and 5.6 in the acacia honey mixture, 35% and 5.4 in the bindweed honey mixture, 45% and 4.7 in the buckwheat honey mixture, 45% and 4.9 in the coffee honey mixture, and 45% and 5.4 in the blended honey mixture, respectively (Tables 1 and 2). These results suggest that pH is not associated with protein aggregation in the upper layer.

**Table 2:** Aggregation pH values.

	pH value^1^	
	Lower layer	Upper layer
Acacia	ND	5.6 ± 0.1
Coffee	5.6 ± 0.1	4.9 ± 0.1
Buckwheat	5.4 ± 0.1	4.7 ± 0.0
Bindweed	5.6 ± 0.0	5.4 ± 0.0
Blended	5.6 ± 0.1	5.4 ± 0.0

1 The pH values are summarized from the results of Figures 2 and 4B.

### 3.6 Association between GA concentration and protein aggregation

The evidence described in this study demonstrates that GA in honey induced protein aggregation in soymilk. To investigate the association between the GA concentration and protein aggregation, the GA concentration in honey was determined (Table 3). The GA concentration was significantly lower in acacia and blended honeys than in bindweed, buckwheat, and coffee honeys (*p* < 0.001). From the GA concentration in honey (Table 3) and the concentration relevant to protein aggregation (Figure 2 and Table 1), the GA concentration for protein aggregation was estimated to be approximately 0.55 mg/mL (0.055%) in the acacia honey mixture, 0.65 mg/mL (0.065%) in the bindweed honey mixture, 0.41 mg/mL (0.041%) in the buckwheat honey mixture, 0.47 mg/mL (0.047%) in the coffee honey mixture, and 0.47 mg/mL (0.047%) in the blended honey mixture. The GDL concentration for tofu preparation is approximately 0.71 mg/mL (0.071%) (Tsukada et al., 2006) or 0.30 mg/mL (0.03%) (Guo and Ono, 2005). Since GDL is hydrolysed to GA in water (Chen et al., 2016), these GA concentrations would be adequate to prepare tofu. The present results strongly suggested that protein aggregation was induced by GA present in the honey. Interestingly, honeys with a high GA content showed comparatively low total sugar content (Table 3), which supports previous reports that GA is derived from glucose (Ruiz-Argüeso and Rodriguez-Navarro, 1973; Ramachandran et al., 2006; Carina et al., 2014).

**Table 3:** Honey gluconic acid concentration and total sugar content.

	GA concentration^1^ (g/L)	Total sugar content^2^ (g/g honey)
Acacia	1.37 ± 0.02^a^	0.71 ± 0.02^a^
Coffee	2.37 ± 0.05^b^	0.65 ± 0.02^a^
Buckwheat	2.03 ± 0.02^c^	0.63 ± 0.01^a^
Bindweed	2.16 ± 0.03^d^	0.66 ± 0.03^a^
Blended	1.34 ± 0.02^a^	0.71 ± 0.06^a^

These values are expressed as means ± SD from three different experiments. Means within the same column bearing different superscripted roman letters are significantly different, with *p* < 0.05 determined by one-way analysis of variance, followed by the Tukey-Kramer test.

1 Gluconic acid (GA) concentrations were analysed using a commercial GA assay kit, as described in the Materials and methods section.

2 Total sugar content was analysed using the phenol-sulphuric acid method.

### 3.7 Association of total sugar content with the aggregation in the upper layer

As shown in Table 2, with all honeys, protein aggregation was induced in the upper layer in the presence of the honey at high concentrations. Protein aggregation in the upper layer was probably caused by a combination of GA and sugar concentrations. The total sugar content tended to be higher in acacia and blended honeys than in bindweed, buckwheat, and coffee honeys. In honeys with high sugar content, little precipitate was observed (Figure 2A) or precipitate was indistinctly observed (Figure 2E). In contrast, in honeys with low sugar content, precipitation occurred largely (Fig. 2B–D). The results strongly suggest that protein aggregation in the upper layer is caused by the combination of GA and sugar concentrations. Besides, kinds of honey with a high sugar concentration had a lower GA concentration. The relationship between sugar content and GA concentration would have resulted from the mechanism underlying GA production, wherein GA is derived from glucose (Ruiz-Argüeso and Rodriguez-Navarro, 1973; Ramachandran et al., 2006).

### 3.8 Reproduction of aggregation behaviours by adding GDL and glucose instead of honey

**Figure 5:**
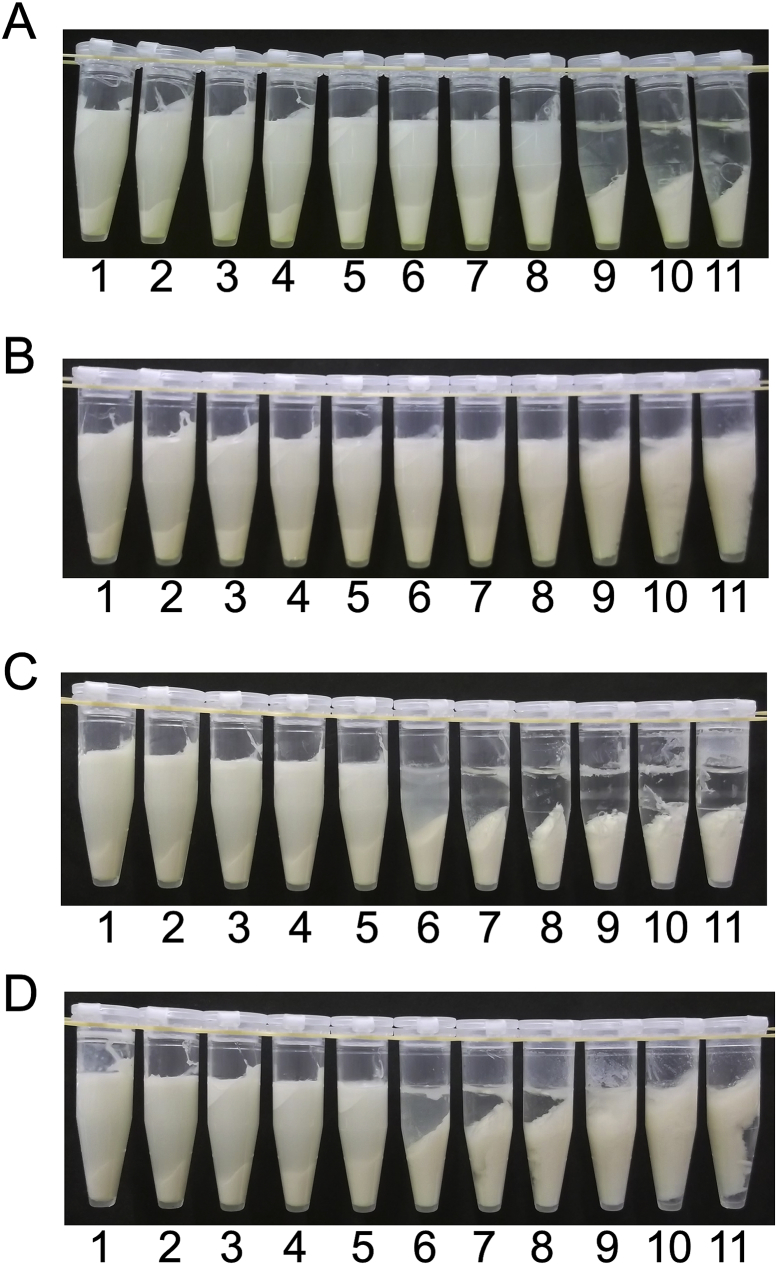
Reproducibility of protein aggregation upon addition of glucono-δ-lactone (GDL)/glucose mixture. For the control, GDL alone was added to an equal volume of soymilk (A and C). As models of acacia honey (B) and coffee honey (D), the GDL/glucose mixture was added to soymilk. (A) The final GDL concentrations were 0.000% (1), 0.015% (2), 0.030% (3), 0.045% (4), 0.060% (5), 0.075% (6), 0.090% (7), 0.105% (8), 0.120% (9), 0.135% (10), and 0.150% (11). (B) The final GDL concentrations are the same as those in panel A. The final glucose concentrations were 0.00% (1), 3.55% (2), 7.10% (3), 10.65% (4), 14.20% (5), 17.75% (6), 21.30% (7), 24.85% (8), 28.40% (9), 31.95% (10), and 35.50% (11). (C) The final GDL concentrations were 0.000% (1), 0.025% (2), 0.050% (3), 0.075% (4), 0.100% (5), 0.125% (6), 0.150% (7), 0.175% (8), 0.200% (9), 0.225% (10), and 0.250% (11). (D) The final GDL concentrations were the same as those in panel C. The final glucose concentrations were 0% (1), 3.25% (2), 6.50% (3), 9.75% (4), 13.00% (5), 16.25% (6), 19.50% (7), 22.75% (8), 26.00% (9), 29.25% (10), and 32.50% (11).

From the results of Figures 2 and 4 and Tables 2 and 3, it was deduced that the protein aggregation in different layers was induced by a different proportion of GA to sugar in honey. To verify the hypothesis, we used GDL/glucose mixtures with the different proportions of GDL to glucose found in pseudo-honey. To test whether the aggregation behaviours of the different honeys were reproducible, GDL/glucose mixtures were added instead of honey (Figure 5). The reason GDL was used is that high-grade commercial GA contains salts, such as magnesium and sodium, which have the potential to enhance precipitation of soymilk proteins (Arii and Takenaka, 2014; Arii and Nishizawa, 2018). In contrast, the influence of salts can be excluded by using GDL, which contains no salts. Protein aggregation was compared between the GDL solutions (Figure 5A and C) and GDL/glucose mixtures (Figure 5B and D). A mixture containing 0.3% GDL and 71% glucose was used as a model of acacia and blended honeys (Figure 5B); a mixture containing 0.5% GDL and 65% glucose was used as a model of bindweed, buckwheat, and coffee honeys (Figure 5D). The glucose concentrations were determined from the results of the total sugar content shown in Table 3. Since the total sugar contents of acacia and blended honeys were determined to be 0.71 g/g honey, a glucose concentration of 71% was used for the model of acacia and blended honeys. Similarly, buckwheat, bindweed, and coffee honeys were approximately 0.65 g/g honey; therefore, their glucose concentration was set to 65%. In addition, 0.3% and 0.5% GDL solutions were also prepared without glucose as controls. These GDL concentration ranges are appropriate for the formation of tofu (Tsukada et al., 2006; Guo and Ono, 2005). In the addition of coffee honey, the GA concentration was estimated to be 0.047% for aggregation from the results of Table 3 and Figure 2. GDL is dynamically changed to GA in the water at 25 °C (Pocker and Green, 1972), and this hydrolysis is increased by heat (Pocker and Green, 1972). In the model experiments, the hydrolysis of GDL to GA was increased by heating at 85 °C. The GDL concentrations in our experiment were selected based on these findings. To test whether GDL alone can induce the protein precipitation in the concentration ranges, 0.3% GDL solution was added to soymilk to have final concentrations in the range of 0.000–0.150% (Figure 5A). Aggregates were observed as a precipitate in above 0.120% GDL concentration. When GDL/glucose solution (0.3% GDL and 71% glucose) with GDL concentration in the same range (0.000–0.150% GDL) was added, the precipitates diminished in the presence of >28.40% glucose (Figure 5B). Precipitates may have been obliterated due to an increase in density by the addition of glucose. In addition, to compare with the model of bindweed, buckwheat, and coffee honeys, 0.5 % GDL solution was added with the final concentration ranging from 0.000-0.250% (Figure 5C). Aggregates were observed in above 0.125% of GDL concentration. When GDL/glucose solution (0.5% GDL and 65% glucose) with GDL concentration in the same range (0.000–0.250% GDL) was added, the precipitate diminished when glucose was >29.25% (Figure 5D). Furthermore, protein aggregation occurred in the upper layer in the presence of 32.50% glucose (Figure 5D). These results indicated that protein aggregation in the upper layer was induced by the combination of specific GDL and glucose concentrations and that the precipitates were suspended at glucose concentrations >28.40%.

### 3.9 Trial product and its texture

**Figure 6:**
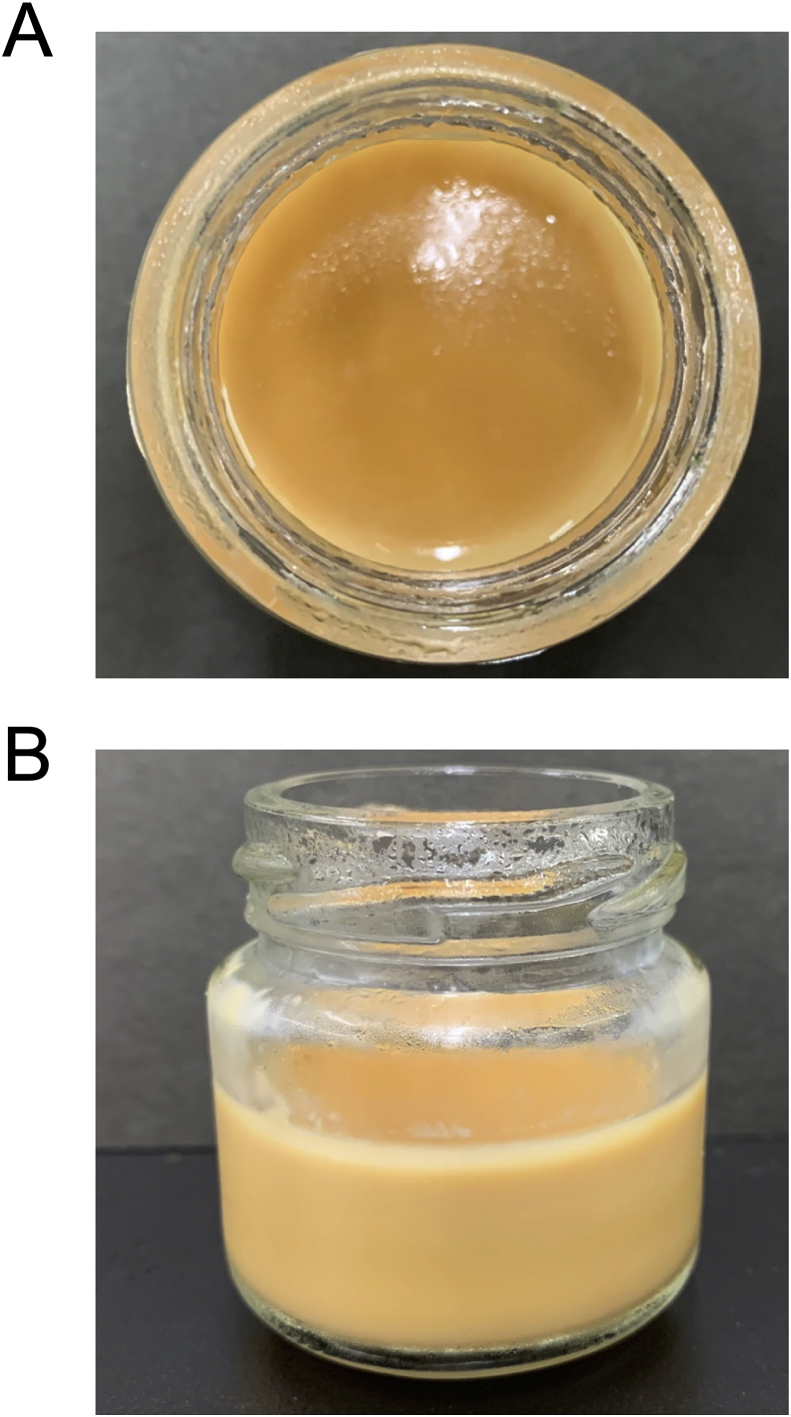
Trial product. Blended honey was mixed with soymilk in a jam bottle. The mixture was steamed, followed by cooling. The product was viewed directly from above (A) and from the side (B).

Our results indicated the possibility that honey could solidify soymilk. We manufactured a trial product using the new honey-mediated aggregation method. Blended honey can be easily obtained in large amounts, which would be economically suitable for the product's initial development. However, blended honey with the lowest GA concentration (according to Table 3) did not show strong soymilk coagulation potential. We, therefore, made a trial product by mixing blended honey, which can be easily obtained in large amounts, with soymilk (honey concentration, (w/w) 43%). As shown in Figure 2E and Table 1, the aggregates were observed in the lower layer with a honey concentration ranging from 35 to 40% and in the upper layer with a honey concentration between 45 and 50%. We considered that the dispersion of aggregates ensured uniform solidification of the trial product. Therefore, the final honey concentration was determined as the average of the highest concentration observed in the lower layer (40%) and the lowest concentration observed in the upper layer (45%). The mixture was poured into a jam bottle and then steamed, followed by cooling down (Figure 6). Hardness, cohesiveness, and adhesiveness of the trial product were 8.44 ± 0.09 (× 10^2^ N/m^2^), 0.56 ± 0.02 and 8.94 ± 2.4 (× 10^2^ J/m^3^), respectively; the thickness of the sample was 16.6 ± 0.3 mm. The values of hardness, cohesiveness, and adhesiveness fell within the range of Licensing Standard III, II, and III, respectively, for food for persons with swallowing difficulties established by the Consumer Affairs Agency of Japan (Consumer Affairs Agency of Japan, 2018). The overall evaluation reflected that the sample adhered to Licensing Standard III, which denoted that the product was pasty. The value for hardness was evaluated to belong to Classification IV of Universal Design Foods, standardized by the Japan Care Food Conference (Fujisaki, 2008). Classification IV denotes that the product can be eaten without masticating. Our results indicated that honey could solidify soymilk to a pasty product.

The combination of GA and sugar concentrations in honey contributes to the adjustment of the sweetness of the product. Briefly, increasing the additive amount of honey brings high sweetness intensity while decreasing the additive amount of honey brings no aggregation. The additive amount of honey and the selection of honey are an essential factor for the development of new products. Thus, it would be easier to adjust the sweetness with honey having a higher GA concentration than with lower GA concentration. Like the presented trial product, we also made other trial products using some kinds of honey (data not shown), and interestingly these had different flavours, tastes, and colours. In other words, the properties of honey exert a considerable influence on the characteristics of the product.

## 4 Conclusions

Our study shows that the addition of honey to soymilk promotes protein aggregation, which can be attributed to the GA content, as well as honey's total glucose content. In acacia and blended honeys, the aggregates were directly observed in the upper layer at higher honey concentrations. In bindweed, buckwheat, and coffee honeys, at an intermediate honey concentration, the aggregates were observed as precipitates, which transferred to the upper layer upon increasing the honey concentration. Considering that GA is derived from glucose in the honey, the assessment of aggregates can be used for simplified discriminant analysis of the quantity of sugar and GA in honey. In a trial product, the mixture of blended honey with soymilk was pasty and indicated that the addition of honey to soymilk could produce new healthy and functional sweets. For the development of such new sweets, further analyses for texture, taste, and processing conditions would be required. Furthermore, the selection of honey would be an essential factor in the development of honey and soymilk-based products. Besides, the different flavours, tastes and colours of the diverse types of honey available worldwide, could be exploited to produce various products with different flavours, tastes, and colours.

## Declarations

### Author contribution statement

Yasuhiro Arii: Conceived and designed the experiments; Performed the experiments; Analyzed and interpreted the data; Contributed reagents, materials, analysis tools or data; Wrote the paper.

Kaho Nishizawa: Performed the experiments; Analyzed and interpreted the data; Contributed reagents, materials, analysis tools or data.

### Funding statement

This research did not receive any specific grant from funding agencies in the public, commercial, or not-for-profit sectors.

### Competing interest statement

The authors declare no conflict of interest.

### Additional information

No additional information is available for this paper.
